# Preventing and managing pharyngocutaneous fistula after total laryngectomy - A narrative review

**DOI:** 10.3389/fonc.2025.1597538

**Published:** 2025-05-21

**Authors:** Cesare Piazza, Claudia Montenegro, Vincent Vander Poorten

**Affiliations:** ^1^ Unit of Otorhinolaryngology – Head and Neck Surgery, ASST Spedali Civili of Brescia, Brescia, Italy; ^2^ Department of Surgical and Medical Specialties, Radiological Sciences, and Public Health (DSMC), University of Brescia, School of Medicine, Brescia, Italy; ^3^ Department of Oncology, Section Head and Neck Oncology, KU Leuven, Leuven, Belgium; ^4^ Otorhinolaryngology-Head and Neck Surgery, Leuven Cancer Institute, University Hospitals Leuven, Leuven, Belgium

**Keywords:** pharyngocutaneous fistula, prevention, management, total laryngectomy, salvage setting, free flap, pedicled flap, salivary bypass tube

## Abstract

Pharyngocutaneous fistula (PCF) remains one of the most frequent and serious complications following total laryngectomy (TL). PCF can lead to severe health issues such as infections and thromboembolic events prolonging hospitalization, as well as to life-threatening large neck vessels blowout and mediastinitis. Despite technical advancements, PCF rate after TL remains around 10%, underlining its challenges in terms of prevention and management. Numerous studies have identified risk factors contributing to PCF development: they can be distinguished into patient-, tumor-, and surgical technique related variables. Nevertheless, a wide consensus has yet to be reached for most of them. Two of the most encountered and recognized risk factors are certainly represented by salvage setting after failure of (C)RT and extension of TL to oro-, hypopharynx or cervical esophagus. In the first scenario, the use of both pedicled and free flaps either with an onlay or an inlay technique have been described, while in case of extended TL, general consensus has been reached in favor of inlay free flaps. Simultaneous use of salivary bypass tube is another commonly applied tool for PCF prevention. This review aims to describe current strategies for prevention and management of PCF after primary and salvage TL with possible extension to adjacent sites.

## 1 Introduction

More than 150 years have passed since the first total laryngectomy (TL) was performed by the Austrian surgeon Theodor Billroth (1). In the twentieth century, advances in anesthesia, surgical sterility, and antibiotic treatments firmly established TL as a key procedure for laryngeal or hypopharyngeal cancers, both in primary and salvage settings after (chemo-)radiation [(C)RT] failure (2, 3). Notwithstanding this, pharyngocutaneous fistula [PCF, already described in the era of Billroth (1)] remains today one of the most frequent and serious complications following TL. Fistula occurs when saliva leaks through the pharyngeal closure line to the neck and skin, usually within the first 7–10 days after surgery (4). The long primary suture of the neopharynx, done within a relatively poorly vascularized tissue, is performed along an always mobile organ full of saliva, secretions, and potentially gastric content, surrounded by a visceral space in which fat tissue, lymph nodes, and part of the musculature have been removed or damaged by the surgical procedure. All these factors can negatively influence the healing process of the neopharynx (4).

PCF can lead to several serious health issues, particularly since it increases the risk of infection of the neck and thromboembolic events, potentially resulting in life-threatening sepsis or mediastinitis. Additionally, it exacerbates malnutrition, carries the risk of cutaneous dehiscence and necrosis, and, in severe cases, exposure of the major blood vessels, with potential catastrophic outcomes such as jugular or carotid blowout (4). From the oncologic point of view, PCF may worsen overall prognosis of advanced tumors by a significant delay in the start of adjuvant treatments when needed.

Surgeons have spent over a century developing surgical techniques to reduce the incidence of PCF (5–9). Despite significant improvements, even in the most skilled hands, the rate of PCF occurrence following primary TL remains around 10% (7, 10, 11), even though reported rates in the literature may vary widely, ranging from 0% (12) to 80% (13). PCF in salvage scenario occurs in up to 44% of the patients and thus is a significant burden to quality of life of these patients (14). PCF prevention could minimize its negative impact and consequences on patients, avoid subsequent surgical treatments, morbidity, and life-threatening complications. Moreover, reducing PCF rates would also reduce one of the most frequent PCF-associated problems which is pharyngo-esophageal stenosis (PES), the latter resulting from the local fibrosis that develops once PCF does heal (3).

This narrative review aims to describe current strategies for prevention and management of PCF after primary and salvage TL for laryngeal or hypopharyngeal cancer.

## 2 Risk factors for PCF

Numerous studies have identified several risk factors contributing to the development of PCF, related to patient, tumor characteristics, and surgical techniques. Nevertheless, a consensus has yet to be reached for most of them.

Patient-related factors primarily involve conditions that impair the healing process, such as smoking and alcohol habits, age, previous history of (C)RT, comorbidities such as diabetes, gastroesophageal reflux, both pre- and postoperative anemia (15), and malnutrition (16–18). A recent study (19) identified postoperative hypothyroidism as a potential contributor to wound complications and PCF. It can be a result from thyroid gland surgical manipulation or hemithyroidectomy and inadequate compensation for the increased postoperative metabolic demand, especially in case of previous RT. In response, levothyroxine supplementation was proposed for patients with high risk of wound complications, leading to a significant reduction in PCF formation and, in case of fistula, a decreased need for surgical intervention with more frequent spontaneous resolution (19).

Tumor-related factors include advanced tumor stage, presence of cervical lymph nodes metastasis and supraglottic subsite [resulting in a relative risk (RR) of 5.96 when a selective neck dissection had to be performed and 1.5 when supraglottic subsite is involved (20)], as well as pharyngeal extension. Patients with hypopharyngeal cancer have a significantly higher rate of PCF formation compared to those with purely intrinsic laryngeal cancer. The University of Brescia group found already in 1999, in a multivariate analysis, a RR of 2.4 for PCF after pharyngolaryngectomy compared to laryngectomy alone without pharyngectomy (21). A recent study confirmed this RR with a PCF rate of 58.9% and 26%, respectively (22).

Postoperative complications can be influenced also by surgical factors including technique employed for pharyngeal repair (type of suture and materials) and surgeon expertise (23–25). The DAHANCA study (23) showed an increased fistula rate in association with a low number of laryngectomies performed by a given surgeon, and an increase after laryngeal preservation strategies. Various surgical techniques had been proposed to reduce the incidence of PCF, but there remains considerable debate over the most effective approach. A recent review (7), however, compared different pharyngeal closure methods and found no significant difference in terms of PCF rates among continuous and T-shape suture, stapler or manual closure, or one-, two-, versus three-layer technique.

Furthermore, additional surgical tricks have been described in the literature aimed at reducing fistula rates, such as the dilute hydrogen peroxide test to detect any leakage along the pharyngeal closure line and allow its immediate intraoperative correction (26), or the application of platelet-rich fibrin during pharyngeal reconstruction (27–29). However, so far, no strong evidence supports their use in clinical practice.

Actually, among all risk factors, only 2 are certainly related to higher risk of PCF occurrence. The first is performing a TL in the salvage setting after failure of (C)RT. Specifically, salvage TL after RT failure alone doubles the risk, while after CRT triples it (11). The second risk factor is the extent of surgical resection (TL extended to oro-, hypopharynx or esophagus) (22, 30–34). In these cases, the importance of adding a flap (either pedicled or free, with onlay or inlay techniques, with or without a salivary bypass tube inside) to the primary suture to lower the risk of PCF is well recognized (3, 15). On the other hand, for all the other potential risk factors (diabetes, chronic renal insufficiency, anemia, malnutrition), we currently lack sufficiently granulated nomograms that can accurately identify patients who would benefit most from this treatment approach.

## 3 Prevention of PCF

### 3.1 Salvage vs primary total laryngectomy

Following the great input given to the non-surgical organ-preservation protocols for laryngeal and hypopharyngeal squamous cell carcinomas (SCC) in the last decades, TL has increasingly been performed as a salvage procedure.

TL performed in a salvage setting is one of the most significant predictive factors for PCF development (35). Liang et al. (36) and Dedivitis et al. (37) reported a PCF rate in salvage TL of 21.2% and 24.6%, respectively, compared to 15.5% in primary TL (37). A PCF rate of 25.5% in salvage TL was also reported more recently by Meulemans et al. (15). Ganly et al. (38) reported a PCF incidence of 15.6% following RT and 31.6% after CRT. Similarly, Sayles et al. (39) reported a PCF rate of 22.8% post-RT and 34.1% post-CRT, confirming that CRT increased the risk of major wound complications, more than RT alone. RT reduces tissue perfusion and oxygenation by obliterative endarteritis, hypoxia, impaired leukocyte migration and fibrosis, and negatively impacts on the healing capabilities of the pharynx (39, 40). On the other hand, chemotherapy increases both local and toxic side-effects of RT.

As a result, to mitigate the risk of PCF in salvage TL, various surgical techniques have been developed. According to the literature, even when there is adequate mucosal tissue available for primary closure of the pharyngeal defect, in the salvage setting it is advisable to routinely utilize a pedicled (PF) or free flap (FF) for pharyngeal closure, with the inlay technique preferred over the onlay approach (41), to enhance the circumference of the pharynx, reduce tension on the suture lines, and provide protection of the pharyngeal sutures by stitching normal mucosa to a well vascularized, healthy, and robust fascio-cutaneous tissue (15, 42–44).

Also in primary TL, the use of a regional flap to protect the suture and potentially minimize the risk of PCF is described. Van Beers et al. (45) suggest the use of a pectoralis major myofascial flap (PMMF) as overlay reinforcement of the pharyngeal closure after primary TL, showing that it significantly reduces the risk of PCF, in particular in patients with low skeletal muscle mass (PCF rate decreased from 31% to 9.9%) (45).

However, in case of primary TL, even though several debated risk factors are considered as critical, no clear evidence supporting the use of vascularized flap is described, except for cases in which mucosa is insufficient for direct closure, such as after resection of oro-and/or hypopharyngeal tumors with possible cervical esophagus extension. As a matter of fact, circumferential and near-circumferential pharyngeal defects are almost always managed with FF, an approach whose benefits are well recognized (46, 47).

### 3.2 Reconstruction after salvage TL

In salvage TL, several surgical strategies have been proposed to prevent PCF, such as the use of revascularized tissues, salivary bypass tube, and secondary delayed tracheoesophageal puncture (TEP) (48–52).

The use of either a pedicled or free flap significantly reduces PCF incidence compared to primary closure, improving wound healing, reducing postoperative inflammation and fibrosis (11, 53, 54). The rational of placing prophylactic well-vascularized flap is to support oxygenation and vascularization of tissue, similarly to the action of hyperbaric oxygen in previously irradiated sites (11). A recent work of Williamson et al. (52) strongly sustains the routine use of vascularized PF or FF in case of salvage TL and the meta-analysis of Sayles and Grant (55) reported a fistula incidence of 10.3% in patients receiving a vascularized tissue flap after salvage TL, a number equal to the 10% rate generally reported in primary laryngectomy.

Flaps can be used as onlay flaps, as a biological reinforcement of the suture line, or with an inlay patch technique sutured to the pharyngeal mucosa. First pedicled flaps (pectoralis major myocutaneous [PMMC] or PMMF) and later on free flaps (anterolateral thigh [ALT] and radial forearm free flaps [RFFF]) have been used in this respect. Righini et al. (56) reported a decreased incidence of PCF from 50% to 23% in TL respectively without and with PMMF used with an onlay technique. Some authors favor PMMF as an onlay flap over a PMMC inlay flap, arguing that PMMC unfavorable thickness hampers matching with the thin hypopharyngeal mucosa (47, 57). In certain studies, onlay PF was associated to lower PCF rates even when compared to onlay or inlay FF (47) and the authors explained this by the non-irradiated origin of the pedicle supplying the vascularization. The Leuven group, however, in a multivariate analysis, found no difference in PCF rate whether using the PM flap as onlay or inlay, but did find a 2.5 RR of PES when using onlay PMMF flaps as opposed to using PMMC as an inlay flap, a logical consequence of the luminal augmentation that the PM skin provides (15).

Also the inlay FF placement for pharyngeal reconstruction is associated with a significant reduction in PCF formation. Moreno et al. reported a PCF incidence of 22.4% in the FF group, followed by 34.5% in the primary closure group and 39.1% in the pedicled flap group (58). The University of Brescia recently reported a PCF rate of 5.4% only in a prospectively recruited series of 55 patients treated by TL after (C)RT failure and reconstructed by inlay ALT or RFFF and use of long-lasting (45 days) salivary bypass tube (50).

A multicenter retrospective review involving 33 institutions and 486 patients (41) who underwent salvage laryngectomy for laryngo-hypopharyngeal SCC, showed that primary closure of the neopharynx was associated with a statistically higher PCF rate and, in case of fistula, higher reoperation rate compared to patients treated by reconstruction with vascularized tissue. It also emphasized that musculo-cutaneous flaps led to worse 12-month speech, nutritional mode, and swallowing scores compared to thinner fascio-cutaneous flaps (41, 59).

A recent inlay FF technique was described by Lin et al. (60) who reported a fistula rate of 10.7%. The procedure required at least 1 cm of edge de-epithelialization at the entire periphery of the fascio-cutaneous FF and a suture in 2 layers: the first layer between the epithelial edge of the flap and the pharyngeal mucosa, while the second between the farthest edge of the flap and the prevertebral fascia.

The field of reconstruction is evolving, with more institutions proactively utilizing FF, which offer lower donor-site morbidity compared to PMMC/PMMF, despite the latter being easier to harvest.

### 3.3 Salivary bypass tube

The use of FF could be optimized by the placement of salivary bypass tube, but heterogeneity of data does not provide strong evidence to support its routine use in clinical practice (61, 62). The salivary bypass tube was placed for the first time in 1978 by Montgomery (63) and it had been recommended in high-risk patients to prevent PCF formation and PES. Literature findings, particularly in high-risk cases such as salvage TL, support its use, with a reported PCF rate of 18.2% (48). Román et al. (64) and Bohlok et al. (65) found that Montgomery salivary bypass tube was related to a decreased risk of PCF, with reported rates of 25% vs. 64.3% (64) and 19% vs. 27% (65), respectively, in patients with and without tube.

Salivary bypass tube was also introduced in the “fistula zero protocol” described by the group from Turin (66) to minimize the rate of PCF formation after TL. They used an Har-El salivary bypass tube (Boston Medical™, Westborough, MA, USA), placed in the neopharynx with a naso-gastric tube positioned inside it and followed by a horizontally continuous barbed suture of the pharynx. In case of salvage setting, a pedicled flap (PMMF) was harvested and placed onlay upon the pharyngeal suture. Crosetti et al. (66) reported PCF in 4 of 77 patients (5.2%), with 1.3% treated by revision surgery and 3.9% managed with a simple compressive dressing.

**Figure 1:**
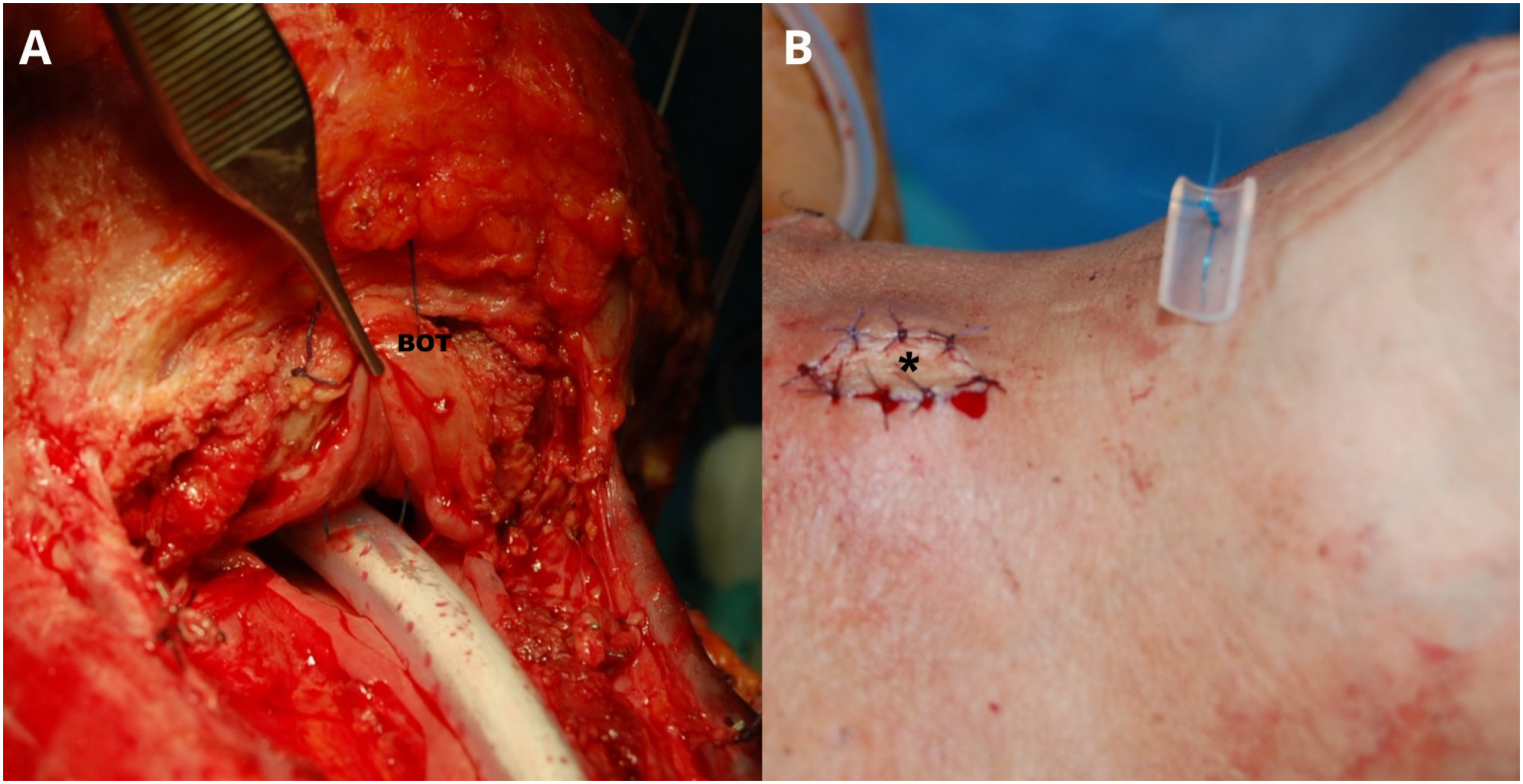
A Montgomery salivary bypass tube (Boston Medical Products, Shrewsbury, MA) is inserted into the surgical defect. Its proximal end is positioned above the suture line at the base of tongue (BOT), while the distal end is placed approximately 4 cm into the distal esophageal stump **(A)**. A nasogastric feeding tube is placed inside the salivary bypass tube. The stent is then secured by stitching it from the BOT to the skin of the chin using a non-absorbable suture **(B)**. Adequate vascularization of the buried free flap is assessed through clinical evaluation of the skin monitor sutured in the middle of the apron flap (asterisk). Patient is usually allowed to eat a semi-liquid diet through the mouth 12–14 days after surgery by removing the nasogastric feeding tube. Patient is discharged with the salivary bypass tube in place for one month more. The stich is cut and the salivary bypass tube removed in the office through the mouth 45 days after surgery.

Even though good results are reported in the literature, there still remains a lack of consensus on the use of salivary bypass tube, its time of removal, and the way of fixing it to avoid its up- or down-ward dislocation. For example, Piazza et al. (46, 50) described their routine use of fixing the salivary bypass tube to the base of tongue and through the skin of chin by a non-resorbable stitch, cut 45 days after surgery (while the patient is allowed to eat a semi-liquid diet per os 12–14 days after surgery), thus removing the stent in the office through the mouth (**Figure 1**). Other individualized surgical and clinical strategies tailored to each patient’s risk profile could be described in the future.

## 4 Treatment of fistula

Although PCF is a common complication following TL, no standardized guideline for its management once occurred has been defined. Fistula often resolves on its own with “spontaneous” healing rates between 80% and 90%, reducing to 44% in previously irradiated necks (21, 67). Consequently, the primary approach to manage this condition involves meticulous conservative care usually consisting of local wound treatment, compressive dressing, antibiotics, and feeding by a nasogastric tube or parenteral nutrition for at least 1–2 weeks. In the literature, the use of modified outside-in EndoVAC therapy is also described as a possible conservative treatment option for PCF. Only few available data so far reported good results, with an appropriate safety profile, especially in case of median PCF not in close proximity with large blood vessels (68). Despite efforts to standardize care, there is no consensus on the appropriate length of conservative treatment, the exact protocols to follow, or clear criteria for determining when the size of a fistula requires conservative management or surgical intervention.

There are cases where surgical intervention is necessary, especially for large and early defects, patients with history of RT or when conservative treatment failed. Surgical options include primary closure or secondary use of well-vascularized tissues, such as PF or FF, despite their higher complication rates and morbidity when compared to their primary application (69, 70).

## 5 Discussion

Despite advances in surgical management and complication prevention, PCF remains one of the most significant challenges following TL even after more than 150 years from its invention, affecting patient recovery, increasing healthcare costs, and diminishing overall quality of life (24). Despite advancements in surgical techniques and postoperative care, the incidence of PCF still remains around 10% after primary procedure, and much higher in the salvage setting, even in the more skilled hands. The analysis of risk factors highlights that PCF formation results from a complex interaction between patient characteristics, surgical procedures, and tumor-related factors. Although numerous predisposing factors have been identified, scientific literature has yet to reach a definitive consensus on many of them. However, there is unanimous agreement on the increased risk of PCF in patients undergoing salvage TL after CRT and in cases of extensive surgical resections involving the oro- or hypopharynx.

The literature offers a range of preventative strategies, including vascularized flap reconstruction, various suture techniques, and salivary bypass tube. However, there is no consensus on a single, universally effective approach for minimizing the risk of PCF, except in the salvage setting where the use of an onlay flap as a support or an inlay flap as augmentation of the pharyngeal closure is widely accepted as effective. The use of PF (such as PMMF) or FF has been shown to significantly reduce PCF incidence, improving neopharyngeal healing through the supply of well-vascularized tissue (11, 41, 49, 53, 54). However, while flap use has become a standard practice in salvage TL to reduce fistula risk, its application in primary procedure for high-risk patients remains a subject of debate and is generally reserved to selected cases.

The use of salivary bypass tube in association with FF is one of the most described preventive tool with the aim of reducing fistula and stenosis, although its effectiveness remains controversial. Some studies report a reduction in PCF rates with use of stent, while others do not find significant differences compared to primary closure without supportive devices (61, 62).

The consequences of PCF can be devastating, increasing the risk of severe infections, PES, malnutrition, and, in the most severe cases, exposure and rupture of major blood vessels. Moreover, it may hamper the oncologic outcomes of patients needing adjuvant treatments by postponing them in a significant and dangerous way. When it occurs, no standardized guideline for its management has been defined. Preventing PCF not only improves surgical outcomes but also has a positive impact on patients’ quality of life by reducing the need for corrective interventions prolonging hospitalization. Future prospective multicentric studies with a large cohort of patients are necessary to develop standardized protocols, such as FF reconstruction and application of salivary bypass tube, to reduce the incidence of PCF.
